# Efficacy of Digestive Endoscope Based on Artificial Intelligence System in Diagnosing Early Esophageal Carcinoma

**DOI:** 10.1155/2022/9018939

**Published:** 2022-06-18

**Authors:** Zhentao Zhao, Meng Li, Ping Liu, Jingfang Yu, Hua Zhao

**Affiliations:** ^1^Endoscopic Diagnosis and Treatment Department, The Second Affiliated Hospital of Shandong University of Traditional Chinese Medicine, 250001 Jinan City, Shandong Province, China; ^2^Office of Invitation to Bid, The Second Affiliated Hospital of Shandong University of Traditional Chinese Medicine, 250001 Jinan City, Shandong Province, China; ^3^Radiology Department, The Second Affiliated Hospital of Shandong University of Traditional Chinese Medicine, 250001 Jinan City, Shandong Province, China; ^4^Department of Spleen, Stomach and Liver Diseases, The Second Affiliated Hospital of Shandong University of Traditional Chinese Medicine, 250001 Jinan City, Shandong Province, China

## Abstract

**Objective:**

To explore the efficacy of digestive endoscopy (DEN) based on artificial intelligence (AI) system in diagnosing early esophageal carcinoma.

**Methods:**

The clinical data of 300 patients with suspected esophageal carcinoma treated in our hospital from January 2018 to January 2020 were retrospectively analyzed; among them, 198 were diagnosed with esophageal carcinoma after pathological examination, and 102 had benign esophageal lesion. An AI system based on convolutional neural network (CNN) was adopted to assess the DEN images of patients with early esophageal carcinoma. A total of 200 patients (148 with early esophageal carcinoma and 52 with benign esophageal lesion) were selected as the learning group for the Inception V3 image classification system to learn; and the rest 100 patients (50 with early esophageal carcinoma and 50 with benign esophageal lesion) were included in the diagnosis group for the Inception V3 system to assist the narrow-band imaging (NBI) with diagnosis. The diagnosis results from Inception V3-assisted NBI were compared with those from imaging physicians, and the diagnostic efficacy diagram was drawn.

**Results:**

The diagnosis rate of AI-NBI was significantly faster than that of physician diagnosis (0.02 ± 0.01 vs. 5.65 ± 0.32 s (mean rate of two physicians), *P* < 0.001); between AI-NBI diagnosis and physician diagnosis, no statistical differences in sensitivity (90.0% vs. 92.0%), specificity (92.0% vs. 94.0%), and accuracy (91.0% vs. 93.0%) were observed (*P* > 0.05); and according to the ROC curves, AUC (95% CI) of AI-NBI diagnosis = 0.910 (0.845-0.975), and AUC (95% CI) of physician diagnosis = 0.930 (0.872-0.988).

**Conclusion:**

CNN-based AI system can assist NBI in screening early esophageal carcinoma, which has a good application prospect in the clinical diagnosis of early esophageal carcinoma.

## 1. Introduction

Esophageal carcinoma is a malignant tumor originating at the mucosal or glandular epithelium of the esophagus [1, 2]. According to epidemiological survey data, the incidence of esophageal carcinoma ranks eighth among all malignant tumors worldwide [3], the incidence in East Asia has been consistently higher than the world average due to the special dietary habits, and in China in 2018, new cases and deaths of esophageal carcinoma accounted for 53.7% and 55.7% of the global total, respectively, with esophageal carcinoma burden about two times the world level [4, 5]. Early diagnosis and treatment is key to reducing such burden in China [6, 7]. Currently, endoscopy, a set of devices for the diagnosis and treatment of digestive diseases by direct acquisition of images of the alimentary tract and digestive organs through the alimentary tract, has become a main method for diagnosis of early esophageal carcinoma in practice. Chromoendoscopy, high-frequency micro probe ultrasonic endoscopy, and electronic staining are common clinical examination modalities. Among them, narrow-band imaging (NBI) is one of the most widely used endoscopic optical staining techniques at present, which, by combining with magnifying endoscopy, can clearly present subtle changes of the capillaries and mucosa within the epithelial papilla with the help of a spectral combination and then effectively improve the detection rate of superficial neoplastic lesions under endoscopy [8], and therefore, it is often used in the clinical diagnosis of early esophageal carcinoma.

**Figure 1:**
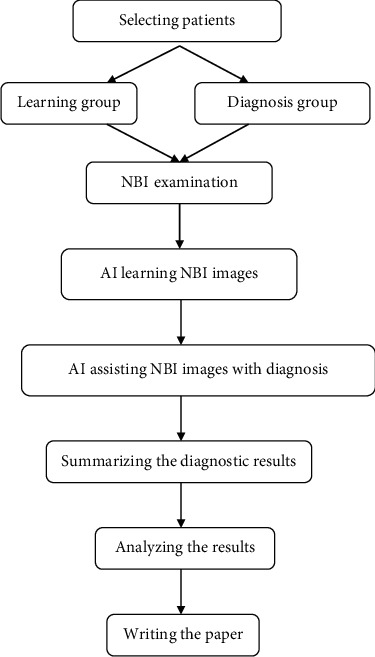
Flow diagram of the study.

In recent years, the development of artificial intelligence (AI) has allowed NBI examinations to be further optimized, and AI model relying on convolutional neural network (CNN) can precisely identify NBI endoscopic images of cancer patients and improve the diagnostic efficiency of NBI [9]. The diagnostic value of the current AI model in gastrointestinal malignancies, such as colorectal cancer and gastric cancer, has been confirmed by literature at home and abroad [10], and Tan et al. reported that the CNN-based AI model had a sensitivity of 90.12% and a specificity of 91.53% for the interpretation of NBI endoscopy in patients with laryngeal cancer [11], with exact application value. Based on this, the study combined AI system with NBI and selected 200 patients (148 with early esophageal carcinoma and 52 with benign esophageal lesion) as the learning group for the Inception V3 image classification system to learn and included another 100 patients (50 with early esophageal carcinoma and 50 with benign esophageal lesion) in the diagnosis group for the Inception V3 system to assist the narrow-band imaging (NBI) with diagnosis, aiming to improve the efficacy of diagnosing early esophageal carcinoma and provide theoretical support for practice and application. The flow diagram of the study is detailed in Figure 1.

## 2. Materials and Methods

### 2.1. Study Design

It was a retrospective study conducted in our hospital from January 2018 to January 2020 to explore the clinical application value of AI system combined with DEN in diagnosing early esophageal carcinoma.

### 2.2. Inclusion and Exclusion Criteria

Inclusion criteria of the study were as follows. (1) The patients were found to have suspected mucosal lesion in the esophagus such as rough, erosive, and mildly protruded mucosa after general white light gastroscopy; (2) the patients were at least 18 years old and voluntarily joined the study; and (3) the patients were treated in our hospital in the whole course and had complete clinical data.

Exclusion criteria of the study were as follows. (1) The patients had clearly diagnosed diseases such as polyp and diverticulum of the esophagus; (2) the patients were in the progressive period of esophageal carcinoma or had accepted treatments such as esophageal carcinoma surgery or chemotherapy before; (3) the patients had obviously extended coagulogram; (4) the patients had severe organic diseases that might affect the accuracy of study results; (5) the patients were pregnant or lactating women; (6) the patients could not tolerate with the NBI examination; and (7) the patients could not communicate with others due to factors such as mental diseases.

### 2.3. General Data

A total of 300 patients with suspected esophageal carcinoma were included in the study; among them, 198 were diagnosed with esophageal carcinoma after pathological examination, and 102 had benign esophageal lesion. A total of 200 patients (148 with early esophageal carcinoma and 52 with benign esophageal lesion) were selected as the learning group for the Inception V3 image classification system to learn; and the rest 100 patients (50 with early esophageal carcinoma and 50 with benign esophageal lesion) were included in the diagnosis group for the Inception V3 system to assist NBI with diagnosis. By collecting the sociodemographic data and clinical manifestation data, it could be concluded that patients with early esophageal carcinoma included in the study (128 males and 70 females) had a mean age of 68.14 ± 9.20 years, and among them, 28 patients had lesion at the median esophagus, 90 patients at the upper esophagus, and 80 patients at the lower esophagus; according to Vienna Classification, 198 patients had high-grade intraepithelial neoplasia (108 with severe atypical hyperplasia and 90 with carcinoma in situ); and the length of patients' lesion was 13.11 ± 2.65 mm. Patients with benign esophageal lesion (72 males and 30 females) had a mean age of 69.17 ± 9.48 years, and according to Vienna Classification, 70 patients had low-grade intraepithelial neoplasia (40 with mild atypical hyperplasia and 30 with moderate atypical hyperplasia), and 32 patients had inflammatory lesions.

### 2.4. Moral Consideration

The study met the principles in *World Medical Association Declaration of Helsinki (2013)* [12] and followed the generally recognized scientific principles and codes of ethics. The patients included understood the study purpose, meaning, contents, and confidentiality and signed the informed consent.

### 2.5. Methods

#### 2.5.1. NBI

The Olympus GIF-H290 endoscopy (Olympus Corporation; NMPA Registration (I) no. 20153223719) and Olympus CV-290SL NBI endoscopy examination system (Olympus Corporation; NMPA Registration (I) no. 20153223192) were adopted. Routine fasting and water deprivation were performed to patients, and 15 min before examination, patients took lidocaine mucilage (Zhejiang Kangde Pharmaceutical Co., Ltd.; NMPA approval no. H20066381), and then, endoscopy examination was performed by endoscopists from the department of digestive medicine with the following steps. Patients were in the left lateral position. First, the scope was entered to the descendant duodenum and then slowly retracted, during which pumping was constantly conducted to expose the gastric cavity and esophageal cavity, and after the scope reached the esophagus, the esophageal mucosa was first observed with white light; when the lesion position was found, it was flushed with dimethyl silicone oil and normal saline, and observation was performed again after removing esophageal mucus. Two physicians used magnifying endoscopy to observe the capillary loops and mucosal microstructure within the papilla of esophageal epithelium to observe the lesion area and then draw corresponding conclusions, which were determined by discussion in case of inconsistency.

#### 2.5.2. Algorithm Construction

The Inception V3 image classification system (Google, California, USA) based on Google Net model was adopted, ImageNet 2012 Challenge training dataset was used, Net was adjusted in NBI image data, algorithm was trained by RMSprop, and the diagnosis model was TensorFlow 1.6. The NBI image data were complete images and marked as pathological benign or malignant for the system to learn, so that the predictive value was infinitely close to the target value, and then, the NBI images of the diagnosis group were interpreted.

### 2.6. Observation Criteria

#### 2.6.1. Diagnosis Results

The diagnosis results from AI-NBI and physicians were recorded, i.e., the number of positive and negative patients obtained by different diagnostic methods.

#### 2.6.2. Diagnostic Efficacy

The diagnostic efficacy of different diagnosis modalities was calculated. (1) Sensitivity: number of true positive cases/(number of true positive cases + number of false negative cases)∗100%; (2) specificity: number of true negative cases/(number of true negative cases + number of false positive cases)∗100%; (3) positive predictive value (PPV): number of true positive cases/(number of true positive cases + number of false positive cases); and (4) negative predictive value (NPV): number of true negative cases/(number of false negative cases + number of true positive cases).

#### 2.6.3. ROC Curve

The ROC curves of the two diagnosis modalities were plotted by recording the positive and negative results from imaging examination into SPSS20.0 and using the ROC analysis method.

### 2.7. Statistical Processing

In this study, the data processing software was SPSS20.0, the picture drawing software was GraphPad Prism 7 (GraphPad Software, San Diego, USA), the item included was enumeration data, the method used was *X*^2^ test, and differences were considered statistically significant at *P* < 0.05.

## 3. Results

### 3.1. Diagnostic Results

The diagnosis rate of AI-NBI was significantly faster than that of physicians (0.02 ± 0.01 vs. 5.65 ± 0.32 s (mean rate of two physicians), *P* < 0.001). See Table 1 for the diagnosis results from AI-NBI and physicians.

### 3.2. Diagnostic Efficacy

Between AI-NBI diagnosis and physician diagnosis, no statistical differences in the sensitivity (90.0% vs. 92.0%), specificity (92.0% vs. 94.0%), and accuracy (91.0% vs. 93.0%) were observed (*P* > 0.05). See Table 2.

### 3.3. ROC Curve

**Figure 2:**
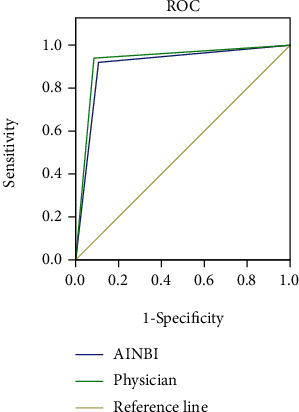
ROC curves of AI-NBI diagnosis and physician diagnosis.

According to the ROC curves, AUC (95% CI) of AI-NBI diagnosis = 0.910 (0.845-0.975), and AUC (95% CI) of physician diagnosis = 0.930 (0.872-0.988), as detailed in Figure 2.

## 4. Discussion

Early esophageal cancer refers to esophageal carcinoma with severe dysplasia, lesions confined within the mucosal layer, and no lymph node metastasis, which can be cured by endoscopic minimally invasive treatment with little trauma, rapid recovery, and a 5-year survival rate more than 90.0% [13], so improving the early detection rate of esophageal carcinoma is key to safeguarding patient outcome. As esophageal carcinoma is mainly squamous cell carcinoma [14], Lugo's solution iodine staining is currently the most common clinical diagnostic method at this stage, which, although has high sensitivity, is limited in clinical application due to its susceptibility to trigger burning sensation and allergic reactions in the stomach [15]. With the continuous advancement of DEN, NBI is developing rapidly, which can apply filters to filter the broad-band spectrum of endoscopic light sources and leave a narrow-band spectrum of green light and blue light and then fully exhibit the subtle changes of the mucosa and the capillaries within the epithelial papilla [16]. When combined with magnifying endoscopy, the contrast of the superficial and underlying blood vessels in the esophageal mucosa can be greatly enhanced, which will facilitate the initial histological diagnosis of early esophageal lesions by clinicians, thereby guiding lesion targeted biopsy and reducing the number of biopsies [17, 18]. Not only that, NBI also has advantages such as easy operation and no adverse reactions induced by chemical stains, so currently, it has been widely used in the clinical diagnosis of Barrett's esophagus early carcinogenesis, early esophageal squamous cell carcinoma, and early esophageal adenocarcinoma.

The report by Liu concluded that the sensitivity and specificity of NBI diagnosing Barrett's esophagus early carcinogenesis were, respectively, 97.0% and 94.0% [19], indicating desirable sensitivity but a certain false positive rate, which was close to the results of Lugo's solution iodine staining. Zhang S.M. et al. showed that the sensitivity of NBI for the diagnosis of esophageal squamous high-grade intraepithelial neoplasia varied greatly, reaching up to 100.0% by experienced endoscopists and only 69.0% by inexperienced physicians [20], indicating that the examination results of NBI are still influenced by the subjective factors of endoscopists. Because clinical upper DEN needs to be done by endoscopists, and diagnosis relies entirely on the endoscopist's visual interpretation and pathological biopsy, the essence is continuously accumulating experience to enhance accuracy. Although endoscopists can fully master the technique of DEN after years of training, the possibility of misdiagnosis and erroneous diagnosis still cannot be excluded. To improve the objectivity and efficiency of diagnosis, AI-assisted DEN has become a hot spot in recent clinical research. AI can enhance its own performance in a way that it learns data without explicit instruction, and feature learning enables AI to actively learn and recognize features in image data, thereby automatically inferring input and output values [21]. On a technical level, such deep learning modality can adopt CNN to analyze complex information, making AI an intelligent system to assist diagnosis, which is beneficial to reduce the study cost of endoscopists, and facilitate the faster application of new technologies such as NBI into practice.

At present, the effectiveness of AI-NBI in the diagnosis of gastrointestinal tumors such as gastric cancer and colorectal cancer has been demonstrated [22]; scholars Barragán-Montero et al. showed that the accuracy of IMRI deep learning-based AI system in diagnosing esophageal cancer was superior to that of 4 endoscopists [23] and that this technique could further determine the depth of invasion of early esophageal carcinoma, proving that AI system can compensate for the shortcomings of incomplete visual capture in humans and assist endoscopists in DEN for precise diagnosis. Scholars Pham et al. constructed an AI model based on CNN and found that its sensitivity for detecting melanoma was 98.0% and the detection rate was significantly higher than endoscopists [24], and this study also found that the diagnosis rate of AI-NBI was 0.02 ± 0.01 s, significantly faster than that of physicians (*P* < 0.001). Zhang S.M. et al. adopted a great number of samples to construct an AI model to learn 8,428 endoscopic images of patients with esophageal carcinoma, and the results showed that the sensitivity of AI diagnosis was 98.0% and the PPV was 40.0% [20]. Based on the features of AI learning, the PPV will continuously increase with the number of samples, and with the building of IOT data platform, the accuracy of AI also elevates. The study showed that the sensitivity, specificity, and accuracy of AI-NBI diagnosis were, respectively, 90.0%, 92.0%, and 91.0%, and AUC (95%CI) = 0.910 (0.845-0.975), while the AUC (95% CI) of physician diagnosis = 0.930 (0.872-0.988), demonstrating that AI system under deep learning could better improve the positive rate of NBI. The future direction should be to achieve real-time diagnosis and make the best use of the advantages of AI technology to reduce the esophageal carcinoma burden in China.

## 5. Conclusion

CNN-based AI system can assist NBI with screening early esophageal carcinoma, presenting a desirable diagnosis rate and a good application prospect in clinical diagnosis of early esophageal carcinoma.

## Figures and Tables

**Table 1:** Analysis of diagnosis results from AI-NBI and physicians.

Group	AI-NBI	Physicians
Sensitivity	90.0 (45/50)	92.0 (46/50)
Specificity	92.0 (46/50)	94.0 (47/50)
Positive predictive value	91.8 (45/49)	93.9 (46/49)
Negative predictive value	90.2 (46/51)	92.2 (47/51)
Accuracy rate	91.0 (91/100)	93.0 (93/100)

**Table 2:** Analysis of diagnostic efficacy of AI-NBI and physicians.

Group	Sensitivity (%)	Specificity (%)	PPV (%)	NPV (%)	Accuracy (%)
AI-NBI	90.0 (45/50)	92.0 (46/50)	91.8 (45/49)	90.2 (46/51)	91.0 (91/100)
Physician diagnosis	92.0 (46/50)	94.0 (47/50)	93.9 (46/49)	92.2 (47/51)	93.0 (93/100)

## Data Availability

Data to support the findings of this study is available on reasonable request from the corresponding author.
